# Terpene constituents of the aerial parts, phenolic content, antibacterial potential, free radical scavenging and antioxidant activity of *Callistemon citrinus* (Curtis) Skeels (Myrtaceae) from Eastern Cape Province of South Africa

**DOI:** 10.1186/s12906-017-1804-2

**Published:** 2017-06-05

**Authors:** Rotimi A. Larayetan, Omobola O. Okoh, Alexander Sadimenko, Anthony I. Okoh

**Affiliations:** 1 Department of Pure and Applied Chemistry, University of Fort Hare, Alice, 5700 South Africa; 2 Chemistry Department, Kogi State University, Anyigba, Kogi Nigeria; 3 SAMRC Microbial Water Quality Monitoring Center, University of Fort Hare, Alice, Eastern Cape South Africa; 4 Applied and Environmental Microbiology Research Group (AEMREG), Department of Biochemistry and Microbiology, University of Fort Hare, Alice, Eastern Cape South Africa

**Keywords:** Volatile oil, Hydro distillation, Antimicrobial, Antioxidant, DPPH, ABTS, Opc

## Abstract

**Background:**

Volatile oil from aromatic plants has been used by ancient Egyptians in embalming for the inhibition of bacterial growth and prevention of decay, *Callistemon citrinus* is used in traditional therapies for the treatment of bronchitis, cough, inflammation and as an antimicrobial herbs. This study examines the essential constituents of the volatile oils obtained from the aerial parts of the plant as well as its antioxidant activity, free radical scavenging, phenolic content and the antibacterial potential of the oils.

**Methods:**

A portion of 500 g, 250 g and 150 g of the leaves, flowers and stems of this plant respectively were subjected to hydro-distillation process for three hours. The oils collected from the various plant parts were immediately subjected to GC-MS analysis. The overall phenolic content of the leaves oil, radical scavenging, antibacterial action and antioxidant activities of the essential oils of both the leaves and flowers of *Callistemon citrinus* were determined using standard methods, with free radical DPPH and ABTS as a reference antioxidant.

**Results:**

Analyses of the three oils revealed a total of twenty-six components for the leaves oil representing 96.84% of the total oil composition, forty-one components for the flowers oil accounting for 98.92% of the whole composition and ten components for the stem oil amounting to 99.98% of the entire oil constituents. The dominant compounds in the leaves oil were eucalyptol (48.98%) and α-terpineol (8.01%), while α-eudesmol (12.93%), caryophyllene (11.89%), (−)-bornyl-acetate (10.02%) and eucalyptol (8.11%) were the main constituents of the flowers oil. In the same vein, the leading constituents in the stems oil were eucalyptol (56.00%) and α-pinene (31.03%). The antioxidant capacities of both the leaves and flowers oils of the plant were evaluated and their IC_50_ were (1.49 and 1.13) for DPPH and (0.14 and 0.03) for ABTS assay respectively. The antibacterial activities of the oils from the (leaves and flowers) were also examined and were found to have wide range of activities against the bacterial strains used in this study.

**Conclusion:**

Observations drawn from this experiment shows clearly that the leaves and flowers of *Callistemon citrinus* possess phenolic compounds and cyclic ether of several pharmacological behaviors.

## Background

Volatile oils are complex and of natural origin having a strong odour, they are usually formed from odoriferous medicinal plants, apart from volatile oil, aromatic plants possess phenolic compounds with numerous pharmacological activities. The study of the constituents of therapeutic plants is of immense significance needed for the production of novel drugs [1].

The genus *Callistemon* belongs to the Myrtaceae family and consists of about thirty-four varieties, ‘Callistemon’ means Anther beauty [2], it is an evergreen shrubs and small tree with lanceolate and aromatic leaves which are prevalent to Australia although a number of them have been brought to other regions like USA [3] and Africa [4]. *Callistemon* is referred to as ‘bottle brush’ owing to its cylindrical brush like flowers resembling the conventional bottle brush. The brilliant red flowers spikes of *Callistemon citrinus* are extremely rich in nectar and as a result attract many birds and insects [5, 6]. *Callistemon* had been used as anticough and antibronchitis in folkloric medicine. The antiphylococcal, nematocidal, larvicidal, pupicidal, antithrombotic and antioxidant activities of the class *Callistemon* species and also the antimicrobial properties of their volatile oils had been widely reported [7].

*Callistemon citrinus* (Curtis) Skeels is a flowering plant and the most commonly cultivated class of the 34 species of *Callistemon* genus [8]. It is a shrub with a height of about 7.5 m tall, having beautiful red flowers with red dark anthers. This plant is used to treat gastrointestinal disorder, pain and infectious diseases caused by bacteria, fungi, virus and other pathogens [9]. The plant is used traditionally in India to combat respiratory conditions like cough, bronchitis and also as insecticides, while its volatile oil is employed as an antimicrobial herbal drug [10, 11]. The leaves are also known for its anti-inflammatory, fungitoxicity; antinociceptive activities [12, 13]. *Callistemon* species are usually grown as decorative plants in houses, offices, streets, and garden area due to its bright red flowers spikes [5, 6]. The essential oil of the leaves has been extensively studied globally by researchers. Eucalyptol (a cyclic monoterpenoid ether) has been found to be the dominant constituents of this plant with various degrees of pharmacological effects and hence it is used as a marker for medicinal essential oil classification [14]. Its medicinal and odoriferous properties are also very unique [15, 16]. Essential oils of the aromatic plants have been used by ancient Egyptians in embalming for the inhibition of bacterial growth and decay. There is a strong in-vitro proof that shows that volatile oils can be active antibacterial agents against several spectrums of pathogenic bacterial strains [17–19], however, there is dearth of information on the chemical profile of the volatile oils of *Callistemon citrinus* obtained from the flowers and stems, thus the present study was undertaken with the aim of investigating the essential constituents of its volatile oil as well as its antioxidant, free radical scavenging, phenolic content and the antibacterial potentials of both the leaves and flowers of *Callistemon citrinus* respectively from Eastern Cape Province of South Africa.

## Methods

### Plant collection

Fresh leaves with flowers were collected in March 2016, from the vicinity of the University of Fort Hare (UFH) Eastern Cape Province of South Africa, the plant was identified by Dr. Meyikeso of the Department of Botany and voucher sample (Larayetan 1) was kept in the Giffen herbarium (UFH), University of Fort Hare, South Africa for record purpose.

### Microbial strains

Pure isolates of *Aeromonas hydrophila (ACC), Escherichia coli (ATCC 35150), Salmonella typhi (ACC), Listeria monocytogenes (ACC), Vibro alginolyticus (DSM 2171), Staphylococcal enteritis (ACC) and Staphylococcus aureus (ACC)* were obtained from Biochemistry and Microbiology Department of the university. All the cultures were maintained on nutrient agar for further use.

### Reagents used

2, 2-azinobis-(3-ethylbenzothiazolin-6-sulfonic acid) diammonium, 2, 2-diphenyl-1-picrylhydrazyl (DPPH) and Potassium persulfate (PPS) were bought from Sigma-Aldrich (St Louis, USA) for this study. All other analytical grade reagents used were sourced from Merck (Germany).

### Quantification of total phenolic content

The overall phenolic content of the leaves volatile oil was determined by the procedure of Folin-Ciocateau. Exactly 1 mL of Folin-Ciocateau reagent was mixed with 1 mL aliquot of the volatile oil and 46 mL of distilled water. After 3 min 3 mL of (2%*w*/*v*) of Na_2_CO_3_ solution was added to the mixture shaken and incubated for two hours in the dark. Absorbance of the resulting mixture was taken on a UV-visible spectrophotometer at 760 nm against the blank and the overall phenolic content was presented as gallic acid equivalent (GAE) in μgmg^−1^ [20].

### In-vitro antioxidant action

#### DPPH assay

The radical scavenging and antioxidant activities of the oils from both the leaves and flowers of the plant were evaluated against the free radical DPPH. Five different concentrations (0.025–0.40 mgmL^−1^) of the oils and commercial antioxidants (β-carotene & Vitamin C) were incubated with a DMSO solution of DPPH for about 30 min at ambient temperature in the dark. The mixture was shaken thoroughly with a vortex machine and the absorbance was taken at 517 nm. The volatile oil ability to scavenge DPPH free radical was calculated using the equation below

$$ \%\mathrm{Inhibition}=\left.\left[\left({A}_{\mathrm{control}}-{A}_{\mathrm{vo}}\right)/{A}_{\mathrm{control}}\right)\right]\times 100 $$

where A_control_ is the absorbance of DPPH + DMSO; A_vo_ is the absorbance of DPPH+ Volatile oil or the commercial antioxidant. The dose response curve was plotted and the IC_50_ value of the commercial antioxidant and volatile oil were calculated [21].

#### ABTS assay

The modified method of Witayapen [22] was used to evaluate the ABTS activity of the volatile oil extracts. The working solution was obtained by oxidation of ABTS stock solution (7 mM) with (2.4 mM) of potassium persulfate in equivalent amounts and the mixture was permitted to react for 12 h at 25 °C. A portion (1 mL) of the resultant solution was further diluted using 60 mL of methanol to obtain an absorbance of 0.706 ± 0.001at 734 nm after 7 min using a UV-spectrophotometer. Summarily, five different concentrations (0.025, 0.05, 0.1, 0.2 and 0.4 mgmL^−1^) of each of the volatile oils were mixed with methanolic solution of ABTS for 7 min at 25 °C in the dark. The absorbance was then measured spectrophotometrically at 734 nm and the % inhibition of ABTS radical by the volatile oils and commercial antioxidants (β-carotene & Vitamin C) was calculated using the equation described for DPPH assay above.

### In vitro antibacterial action

Antibacterial activity of the volatile oils was tested by means of the agar well diffusion method as described by Collin [23]. The microbial cultures used in this study were inoculated in nutrient broth (Oxoid) and incubated for 24 h at 37 ± 0.1 °C. Sufficient amount of Muller Hilton Agar (Oxoid) were poured into sterile petri dishes and permitted to solidify under aseptic situation. Using a sterilized cork borer, five 6 mm diameter wells were evenly distributed in freshly prepared and solidified Mueller Hilton agar (Oxoid) in petri dishes. The bacterial culture was adjusted to 0.5 Mc Farland turbidity standard and the test microbes (0.1 mL) were inoculated with a germ-free swab on the exterior of the appropriate solid medium in each of the petri dishes. Varying concentrations of the volatile oil made from the stock ranging from 0.04 to 0.025mgmL^−1^ were prepared and introduced into each of the wells and labelled appropriately. The inoculated petri dishes were incubated at 37 °C for 24 h. All the petri dishes were then examined for zones of growth inhibition surrounding the individual wells and the average diameter of these zones was measured in millimeters. All tests were performed under hygienic conditions.

### Separation of volatile oils

Five hundred grams (500 g) of the leaves, two hundred and fifty grams (250 g) of the flowers and one hundred and fifty grams (150 g) of the stems of this plant were sequentially subjected to hydro- distillation process for three hours in Clevenger apparatus in accordance with the European Pharmacopoeia (2004) [24]. The volatile oil was consecutively extracted using 7 L, 4 L and 2 L of water. The oils collected from the various plant parts were immediately subjected to GC-MS analyses.

### Gas chromatography- mass spectrometry (GC-MS)

GC-MS analyses were ran on a Hewlett-Packed HP 5973 mass spectrometer connected with an HP-6890 gas chromatograph. Operating conditions for this analyses were as followed: column temperature (original temperature) -70 °C, (highest temperature) -240 °C, (equilibration time) -3 min, (ramp) -4 °C min^−1^, (concluding temperature) -240 °C; inlet mode: split less, initial temperature 220 °C, pressure 8.27 psi, flush out flow 30 mL/min, flush out time 0.20 min, gas brand: helium, column: capillary, 30 m × 0.25 mm i.d., coat thickness 0.25 μm, original flow 0.7 mL/min, linear velocity 32 cm/s; MS: EI method at 70 eV.

### Detection of components

Chemical components of these oils were identified on the basis of their individual retention times with a reference to homologues series of n-alkanes in the robust NIST Library 2014. The mass spectra fragmentations of the compounds were compared to the available data [25–27].

### Statistical analysis

Statistical analyses were carried out using Microsoft Excel 2007.

## Results

### Discussion

#### Constituents of the volatile oils

Hydro distillation of the leaves, flowers and stems of *Callistemon citrinus* produced a clear light, pale yellow and light oil with percentage yields of 0.70% (leaves), 0.80% (flowers) and 0.50% (stems) *v*/*w* of the wet samples. The identified components, retention times and percentage compositions of the chemical compounds are given in Table 1 above. From the table it is obvious that qualitative and quantitative constituents of the oils are different from each other.

**Table 1:** Fractional compositions of constituents of the leaves flowers and stems oil of *Callistemon citrinus*

Retention Time (Min)	Constituents	Oil Composition (%)		
		Leaves	Flowers	Stems
3.43	Isopentyl Acetate	0.17	-	-
3.89	β-Thujene	-	0.65	-
3.97	α-Pinene	20.02		31.03
3.98	1R-α-Pinene	-	4.57	-
4.12	Camphene	0.63	3.81	-
4.25	Vinyl Amyl Carbinol	-	0.14	-
4.29	β-Phellandrene	-	0.37	-
4.34	β-Pinene	1.10	2.26	1.18
4.52	Heptane-3,4-dimethyl	0.81	-	-
4.69	o-Cymene	-	1.22	-
4.73	Limonene	-	-	3.97
4.77	Eucalyptol	48.98	8.11	56.00
4.97	γ-Terpinene	0.21	1.76	-
5.21	3-Carene	0.17	0.05	-
5.23	β-linalool	0.57	-	1.08
5.44	α-Fenchol	0.93	-	-
5.67	Pinocarveol	5.75	-	0.51
5.73	(+)-2-Bornanone	-	4.35	-
5.76	Camphenilanol	0.27	-	-
5.85	Pinocarvone	2.81	-	-
5.88	Borneol	-	1.75	-
5.94	Terpinen-4-ol	0.79	0.77	0.77
6.03	α-Terpineol	8.01	0.22	3.69
6.09	Myrtenol	0.29	-	-
6.22	Cis-Carveol	0.70	-	-
6.23	(+)-Camphene	-	0.26	-
6.30	Cis-p-metha-1(7), 8-diene-2-ol	0.53	-	-
6.42	Geraniol	0.40	-	-
6.75	(−)-Bornylacetate	-	10.02	-
7.11	Trans −2-acetoxyl-1,8-cineole	0.18	-	-
7.23	4-Carene	0.76	-	-
7.42	Copaene	-	0.34	-
7.77	Caryophyllene	-	11.89	-
7.89	Aromadendrene	-	3.80	-
7.93	α-Gurjinene	-	0.70	-
7.98	Humulene	-	1.05	-
8.03	Alloaromadendrene	-	0.89	-
8.08	α-Elemene	-	0.51	-
8.16	α-Farnesene	-	0.42	-
8.25	Bicyclogermacrene	-	5.71	-
8.36	(+)-δ-Cadinene	-	2.10	-
8.52	Elemol	-	0.58	-
8.65	(+)-Valencene	-	0.37	-
8.76	Spathulenol	0.19	3.16	1.10
8.80	(−)-Globulol	0.39	-	0.65
8.82	(+)-Ledene	-	2.33	-
8.86	γ-Gurjinene	0.20	-	-
8.87	(+)-Viridiflorol	-	0.49	-
8.91	Rosifoliol	-	0.50	-
8.96	Benzoic acid, 3,4-dimethoxy, methyl Ester	1.72	-	-
9.02	β-Eudesmol	0.26	-	-
9.07	γ-Eudesmol	-	5.21	-
9.11	Hinesol	-	2.03	-
9.22	α-Eudesmol	-	12.93	-
9.28	Cis-1(7), 8-p-mentha-diene-2-ol	-	1.05	-
9.38	5-Amino-1-phenylpyrazole	-	1.15	-
9.52	p-Cymene-3-propionic acid, α-methyl	-	0.35	-
9.82	3-Carene, 4-isopropenyl	-	0.34	-
9.93	Cis-Calemenene	-	0.42	-
10.69	Geranyl-α-Terpinene	-	0.28	-
	Total	96.84	98.92	99.98
	Hydrocarbons Monoterpene	22.89	15.24	36.18
	Oxygenated Monoterpenes	70.01	26.27	62.05
	Sesquiterpene Hydrocarbons	0.20	30.53	-
	Oxygenated Sesquiterpenes	0.84	24.90	1.75
	Others	2.88	1.98	-

Twenty-six components for the leaves oil amounting to 96.84%, forty-one components for the flowers oil representing 98.92% and ten components for the stems oil amounting to 99.98% were identified in the three oil samples. The leaves oil was composed of mainly oxygenated monoterpenes (70.01%), followed by monoterpene hydrocarbons (22.89%), sesquiterpene hydrocarbon (0.20%), and oxygenated sesquiterpenes (0.84%). The dominant constituents in the leaves oil samples were eucalyptol (48.98%), α-Pinene (20.02%), α-terpineol (8.01%) and pinocarveol (5.75%). Other notable components found were Pinocarvone (2.81%) and β-pinene (1.10%). Minor constituents include α-fenchol (0.93%), terpinen-4-ol (0.79%), camphene (0.63%) and β-linalool (0.57%).

The flower oil was found rich in sesquiterpene hydrocarbons (30.53%), followed by oxygenated monoterpenes (26.27%), oxygenated sesquiterpenes (24.90%) and monoterpenes hydrocarbon (15.24%). The main constituents characterizing the floral oil were α-eudesmol (12.93%), caryophyllene (11.89%), (−)-bornyl acetate (10.02%), eucalyptol (8.11%), bicyclogermacrene (5.71%), γ-eudesmol (5.21%) and 1R-α-Pinene (4.57%).

Similarly, the stem oil comprises of oxygenated monoterpenes (62.05%) and monoterpene hydrocarbons (36.18%). The key components dominating the stem oil were eucalyptol (56.00%), α-pinene (31.03%), limonene (3.97%) and α-terpineol (3.69%).

The various components of volatile oil have been associated with different therapeutic activities. Eucalyptol, which is the chief constituents of both leaves and stems oils of *Callistemon citrinus* in addition to its anti-inflammatory activity possesses notable antiviral activity, antitussive, bronchodilator, mucolytic and mucociliary effects. It also has positive activity on the lung function for the ordinary cold or persistent obstructive pulmonary ailment [28]. It also possesses bacteriostatic and bactericidal activity [29].

α-pinene which ranks as the next key constituent of the leaves oil in this work reportedly has biological activity with broad spectrum that is anti-inflammatory [30], antibacterial [31], antioxidant [32], anticancer [33] and antinociceptive [34]. Due to these properties stated above the plant has been found useful in the traditional management of some infectious diseases caused by bacteria, fungi and virus. It is also used in the treatment of bronchitis and respiratory conditions like cough.

The flower volatile oil of our sample has α-Eudesmol as the major component. It does show high voltage-gate calcium channel blocker activity, which is a foremost problem in anti-migraine treatment [35] and is available to attenuate post-ischemic brain injury in rats [36].

Volatile oil of the leaves of *Callistemon citrinus* in this study showed similar profile compared to those reported earlier in literature as shown in (Table 2) above [12, 37–42]. Eucalyptol was found to be the main constituent though in varying amount except for Himalaya where the dominant component of the leaves oil was α-pinene (32.30%) but the flowers oil from this region was still eucalyptol. This could be attributed to environmental factors like variable ecological and climatic conditions in the regions, as well as the nature of the plant and the processing method [43].

**Table 2:** Major components of volatile oil of *Callistemon citrinus* from various part of the world

Origin		Major Components	Reference
South(f) Africa		α-Eudesmol (12.93%), Caryophyllene (11.89%), Bornyl-acetate (10.02%), Eucalyptol (8.11%), Bicyclogermacrene (5.71%) and γ-Eudesmol (5.21%).	This Present Study
South(l) Africa		Eucalyptol (48.98%), α-Pinene (20.02%), α-Terpineol (8.10%), Isopinocarveol (5.75%) and Pinocarvone (2.81%).	This Present Study
South(s) Africa		Eucalyptol (56.00) and α-Pinene (31.03).	This Present Study
Himalaya(f)		Eucalyptol (36.6%), α-Pinene (29.7%).	[37]
Himalaya (l)		α-Pinene (32.30%), Limonene (13.1%), α-Terpineol (14.6%)	[37]
Iran (l)		Eucalyptol (34.20%), α-Pinene (29.0%), α-Terpineol (16.70%), α-Phellandrene (9.0%).	[38]
Iran	(l)	Eucalyptol (67.60%), α-Pinene (9.40%), β-Pinene (4.70%)	[39]
	(f)	α-Pinene (25.70%), eucalyptol (18.10%), β-Pinene (7.30%), Linalool (5.30%).	[39]
	(s)	Eucalyptol (41.30%), α-Pinene (19.10%), α-Terpineol (4.10%),	[39]
Reunion Island (l)		Eucalyptol (68.0%), α-Pinene (12.80%), α-Terpineol (10.6%).	[40]
Lower(l) Region of Himalaya		Eucalyptol (66.30%), α-Pinene (18.70%)	[41]
South Africa (l)		Eucalyptol (61.20%), α-Pinene (13.40%), β-Pinene (4.70%)	[12]
Pakistan		Eucalyptol^a^, α-Terpineol^a^	[42]

*l* leaves, *f* flowers, *s* stems, ^a^Quantitative data not available

α- eudesmol (12.93%), caryophyllene (11.89%) and bornyl acetate (10.02%) were the dominant components found in the volatile oil of the flowers of this plant under investigation. Eucalyptol was also notably present in the flower oil but the content was low (8.11%) compared to the stems and leaves oils which were (56.00%) and (48.98%) respectively. The oil collected from the flowers shows no homogeneity in constituents in comparison with others in the literature (Table 2). While the flowers essential oil from Iran showed α-pinene (25.70%) and eucalyptol (18.10%) as the principal components [39], another from Himalaya recorded eucalyptol (36.60%) and α-pinene (29.70%) as its foremost components [37]. The present study reveals that α- eudesmol (12.93%), caryophyllene (11.89%) and bornyl acetate (10.02%) are the major constituents in the flower oil. This might be related to the effects of several factors like relative humidity, irradiance, photoperiod, method of extraction, plant cultivation techniques, soil structure and climate which could greatly influence the composition and quality of essential oil [44].

#### Overall phenolic content (Opc)

The overall phenolic content in the leaves volatile oil was found to be 899.00 μgmg^−1^ gallic acid equivalent, which was higher than the same species from India that recorded 261 mg/g [45]. This shows the presence of phenolic compounds such as α-terpineol and pinocarveol in the leaves oil. Phenolic compounds in plants are known to increase its antioxidant activity [46]. They have a major responsibility in scavenging free radicals that cause oxidative stress due to their antioxidant capacity against peroxyl radicals. This helps them scavenge electrophiles and active oxygen species, limit auto-oxidation by chelating metal ions and increase the ability to adjust some enzymes action [47].

#### Antibacterial activities of the leaves and flowers volatile oil

**Fig. 1:**
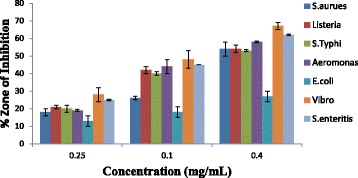
Antibacterial activity of leaves oil of *Callistemon citrinus*

**Fig. 2:**
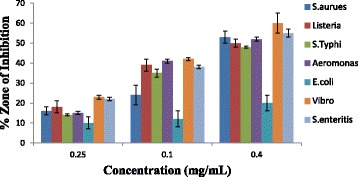
Antibacterial activity of flower oil of *Callistemon citrinus*

The present work examined the in-vitro antibacterial activities of the volatile oils from leaves and flowers of *Callistemon citrinus* on the four gram negative and three gram positive bacteria as shown in (Table 3). The activities of the oils in terms of inhibitory zones were effective on all the tested bacteria showing that the plant under study has very wide spectrum of action against both gram positive and gram negative bacteria (Figs. 1 & 2). The inhibitory effect of both the leaves and flowers oils was highest against gram negative bacteria like *Vibro alginolyticus DSM 2171* (67 ± 2.0 & 60 ± 5.0 mm) *and Aeromonas hydrophila ACC (*58 ± 0.3 & 52 ± 1.0 mm), as well as the gram positive bacteria such as *Staphylococca*l *enteritis ACC* (62 ± 0.5 & 55 ± 2.0 mm) at a concentration of 0.4 mg/mL, while the lowest effect was recorded for *E.coli ATCC 35150* (27 ± 3.0 & 20 ± 4.0 mm). Both the leaves and flowers oil showed highest inhibitory effect on gram negative bacteria which was contrary to some reports published in literature [48]. The antibacterial properties of these volatile oils were found comparable to that of the leaves volatile oil from Western part of South Africa which gave inhibition zone ranging between 13.3 and 26.3 mm for *S. aureus* (ATCC 3983), a gram positive bacteria, also *E.coli* (ATCC 4983) *and P. aeruginosa* (ATCC 7700) which are gram negative bacteria [12]. The antibacterial effect observed in this plant may be linked to some bioactive compounds such as alkaloids, tannins, terpenoids, ether and phenolic compounds like flavonoids, which are considered to be bacteriostatic and fungistatic [49, 50]. This effect correlates with its folkloric uses and shows that it is an efficient antimicrobial plant that can be employed in alternative medicine for the treatment of bacterial infection [51].

**Table 3:** Inhibition zone (mm) showing antibacterial activities of volatile oils and ciprofloxacin against bacterial test organisms

Microorganism	Positive control			Zone of inhibition of volatile oils and standard drug					
Concentration	Ciprofloxacin (mgmL^-1^)			Leaves volatile oil (mgmL^-1^)			Flowers volatile oil (mgmL^-1^)		
	0.4	0.1	0.025	0.4	0.1	0.025	0.4	0.1	0.025
Gram negative Bacteria									
*Aeromonas hydrophilia ACC*	34.0 ± 0.6	26.0 ± 2.0	20.0 ± 0.5	58.0 ± 0.3	44.0 ± 4.0	19.0 ± 0.6	52.0 ± 1.0	41.0 ± 1.0	15.0 ± 0.8
*Escherichia coli ATCC 35150*	38.0 ± 4.0	32.0 ± 4.0	24.0 ± 0.9	27.0 ± 3.0	18.0 ± 3.0	13.0 ± 3.0	20.0 ± 4.0	12.0 ± 4.0	10.0 ± 3.0
*Vibro alginolyticus DSM 2171*	33.0 ± 2.0	29.0 ± 2.0	23.0 ± 0.4	67.0 ± 2.0	48.0 ± 5.0	28.0 ± 4.0	60.0 ± 5.0	42.0 ± 0.8	23.0 ± 1.0
*Salmonella typhi ACC*	40.0 ± 1.0	34.0 ± 0.6	28.0 ± 3.0	53.0 ± 0.5	40.0 ± l.0	20.0 ± 2.0	48.0 ± 0.5	35.0 ± 2.0	14.0 ± 0.5
Gram positive Bacteria									
*Staphylococca*l *enteritis ACC*	40.0 ± 5.0	32.0 ± 1.0	28.0 ± 0.2	62.0 ± 0.5	45.0 ± 0.1	25.0 ± 0.4	55.0 ± 2.0	38.0 ± 0.9	22.0 ± 0.9
*Staphylococcus aureus ACC*	29.0 ± 2.0	24.0 ± 0.0	17.0 ± 2.0	54.0 ± 4.0	26.0 ± 1.0	18.0 ± 2.0	53.0 ± 3.0	24.0 ± 5.0	16.0 ± 2.0
*Listeria monocytogenes ACC*	44.0 ± 6.0	38.0 ± 0.2	32.0 ± 1.0	54.0 ± 2.0	42.0 ± 2.0	21.0 ± 1.0	50.0 ± 2.0	39.0 ± 3.0	18.0 ± 3.0

Zone of inhibition (millimeter), *ACC* Aemreg culture collection, *ATCC* American type collection center, values are mean ± SD, *n* = 3

#### In-vitro antioxidant action

The in-vitro antioxidant activities of the volatile oils were evaluated using DPPH radical scavenging test. The violet colour production of DPPH dissolved in DMSO is due to its unpaired nitrogen electrons. The DPPH radical is in the process reduced to DPPH-H, turning from violet to yellow in the presence of antioxidant compound [52].

**Fig. 3:**
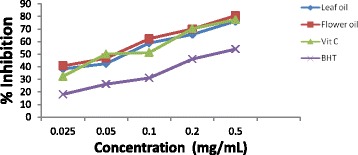
DPPH scavenging action

**Fig. 4:**
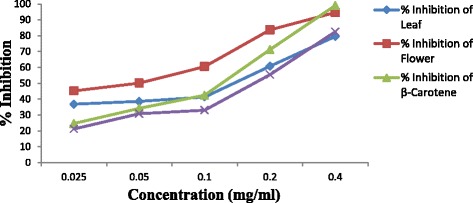
ABTS scavenging action

The inhibition of the DPPH radical by the volatile oil of the leaves and flowers was concentration dependent. The inhibition percentage of the volatile oils at different concentrations (0.025, 0.05, 0.1, 0.2, 0.4 mgmL^-1^) ranged between 38.3% and 76.2% for the leaves oil and from 40.7% to 80.6% for the flowers oil. In the same vein, the percentage of inhibition for both the ascorbic acid and β-carotene varied as 18.1%–54.04% and 32.4% –77.45% respectively (Fig. 3). The leaves and the flowers oils were capable of reducing the DPPH radical by 50% with IC_50_ of 1.49 and 1.13 mgmL^-1^ compared to β-carotene and ascorbic acid which have an IC_50_ of 1.28 and 3.57 mgmL^-1^ (Table 4). The capacity of the DPPH radical scavenging of the flowers oil in terms of percentage inhibition and IC_50_ was higher than those of the leaves oil and the two synthetic antioxidant drugs. The inhibition of DPPH free radical by both the leaves and flowers oils were higher than that reported for the volatile oil of same species in Iran [39]. For the ABTS assay, the essential oils collected from the leaves and flowers of the plant under study showed free radical scavenging activities which were dose dependent, having a maximum activity of 79.47% at 0.4 mgmL^-1^ for the leaves oil and 95.61% for the flowers oils (Fig. 4). The oils from both plant parts showed a 50% reduction of 0.14 and 0.03 mgmL^−1^ respectively which implies that the flowers oil possesses higher antioxidant capacity than the leaves oil and other typical antioxidants (Vitamin C & BHT) with IC_50_ of 0.13 and 0.19 respectively (Table 4). The high scavenging activity of the leaves oil over BHT (standard antioxidant) could be due to the high content of Eucalyptol in the leaves oil [53].

**Table 4:** IC_50_ profile of the leaves and flowers oil of *Callistemon citrinus* (mgmL^−1^)

		*Callistemon citrinus*		Standard Antioxidant (Positive control)		
S/N	Activity	Leaves oil (IC_50_)	Flowers oil (IC_50_)	Vitamin C (IC_50_)	β-Carotene (IC_50_)	BHT (IC_50_)
1	DPPH	1.49	1.13	3.57	1.28	-
2	ABTS	0.14	0.03	0.13	-	0.19

*DPPH* 2,2-diphenylpicrylhydrazyl radicals, *ABTS* 2,2′-azino-bis diammonium salt, *BHT* Butylated hydroxyl toluene

The antioxidant activities of volatile oils are reportedly not only due to phenolic content of the oil but also some other constituents like monoterpene alcohol, ketone, aldehyde, hydrocarbons and ethers which are known to contribute to the free radical scavenging activity of some volatile oil [54]. Volatile oils of *Thymus caespititus, Thyme camphorates,* and *Thyme mastichina* which contain high contents of linalool and eucalyptol were found with high antioxidant activities which were almost equal to that of α-tocopherol [55]. Similarly, the *M. aquatic* high scavenging activity was due to the presence of eucalyptol in the volatile oil [53]. The essential oil of the plant in the present study also showed high level of eucalyptol (monoterpenoid ether) which might be responsible for its antioxidant activity.

## Conclusion

This study represents the first analyses of the volatile constituents of the essential oils from *Callistemon citrinus* leaves, flowers and stem to the best of my knowledge in the Eastern Province of South Africa. Aside from the traditional uses of the extract of the plant, its volatile oil possesses high-quality antioxidant potential and may possibly compete well with synthetic antioxidant drugs in the market. Observation drawn from this experiment shows clearly that the leaves and flowers of *Callistemon citrinus* possess substantial quantity of the phenolic compounds and cyclic ether with several pharmacological behaviors. The present investigation showed that the studied plant is a good traditional herb of potential value for the cure of various ailments.

## Abbreviations

ABTS^*+^: 2, 2-azinobis-(3-ethylbenzothiazolin-6-sulfonic acid) diammonium salt radical
ACC: AEMREG culture collection
ATCC: American type collection center
BHT: Butylated hydroxyl toluene
DMSO: Dimethyl sulphur oxide
DPPH*: 2, 2-diphenyl-1-picrylhydrazyl
f: flowers
GC-MS: Gas chromatography-mass spectrometry
IC_50_: Inhibitory concentration at 50%
l: leaves
OPC: Overall phenolic content
PPS: Potassium persulfate
s: stems
